# Acute Blood Pressure Responses in Healthy Adults During Controlled Air Pollution Exposures

**DOI:** 10.1289/ehp.7785

**Published:** 2005-05-19

**Authors:** Bruce Urch, Frances Silverman, Paul Corey, Jeffrey R. Brook, Karl Z. Lukic, Sanjay Rajagopalan, Robert D. Brook

**Affiliations:** 1 Gage Occupational and Environmental Health Unit, St. Michael’s Hospital, Toronto, Ontario, Canada; 2 Department of Public Health Sciences,; 3 Department of Medicine, and; 4 Department of Chemical Engineering, University of Toronto, Toronto, Ontario, Canada; 5 Air Quality Research Branch, Meteorological Service of Canada, Environment Canada, Toronto, Ontario, Canada; 6 Mount Sinai School of Medicine, New York, New York, USA; 7 Department of Internal Medicine, University of Michigan, Ann Arbor, Michigan, USA

**Keywords:** air pollution, blood pressure, hypertension, ozone, particulate matter, PM_2.5_

## Abstract

Exposure to air pollution has been shown to cause arterial vasoconstriction and alter autonomic balance. Because these biologic responses may influence systemic hemodynamics, we investigated the effect of air pollution on blood pressure (BP). Responses during 2-hr exposures to concentrated ambient fine particles (particulate matter < 2.5 μm in aerodynamic diameter; PM_2.5_) plus ozone (CAP+O_3_) were compared with those of particle-free air (PFA) in 23 normotensive, non-smoking healthy adults. Mean concentrations of PM_2.5_ were 147 ± 27 versus 2 ± 2 μg/m^3^, respectively, and those of O_3_ were 121 ± 3 versus 8 ± 5 ppb, respectively (*p* < 0.0001 for both). A significant increase in diastolic BP (DBP) was observed at 2 hr of CAP+O_3_ [median change, 6 mm Hg (9.3%); binomial 95% confidence interval (CI), 0 to 11; *p* = 0.013, Wilcoxon signed rank test] above the 0-hr value. This increase was significantly different (*p* = 0.017, unadjusted for basal BP) from the small 2-hr change during PFA (median change, 1 mm Hg; 95% CI, −2 to 4; *p* = 0.24). This prompted further investigation of the CAP+O_3_ response, which showed a strong association between the 2-hr change in DBP (and mean arterial pressure) and the concentration of the organic carbon fraction of PM_2.5_ (*r* = 0.53, *p* < 0.01; *r* = 0.56, *p* < 0.01, respectively) but not with total PM_2.5_ mass (*r* ≤ 0.25, *p* ≥ 0.27). These findings suggest that exposure to environmentally relevant concentrations of PM_2.5_ and O_3_ rapidly increases DBP. The magnitude of BP change is associated with the PM_2.5_ carbon content. Exposure to vehicular traffic may provide a common link between our observations and previous studies in which traffic exposure was identified as a potential risk factor for cardiovascular disease.

Exposure to fine particulate air pollution [aerodynamic diameter < 2.5 μm (PM_2.5_)] is associated with increased cardiopulmonary mortality (Pope et al. 2002; Samet et al. 2000). Coronary ischemic events, occurring as rapidly as 1–2 hr after exposure, account for a major portion of this heightened mortality (Peters et al. 2001, 2004; Pope et al. 2004). In addition, an enhanced risk for acute cerebrovascular strokes has been linked to particulate air pollution (Hong et al. 2002; Tsai et al. 2003).

Several biologic mechanisms have been demonstrated that may in part explain these findings (Brook et al. 2003), including acute arterial vasoconstriction after exposure to concentrated ambient fine particles (CAP) with added ozone (Brook et al. 2002). In the latter study (Brook et al. 2002), subjects exposed to CAP with a higher organic carbon content had greater vasoconstriction than did those subjects exposed to CAP with less organic carbon content (Urch et al. 2004). Considering that PM_2.5_ has also been shown to alter autonomic balance (Devlin et al. 2003; Gold et al. 2000; Magari et al. 2001), it is reasonable to hypothesize that air pollution exposure can meaningfully affect blood pressure (BP). Using a randomized, sham [particle-free air (PFA) without added O_3_] controlled study, we investigated the effect of short-term inhalation of CAP with added O_3_ (CAP+O_3_) on BP and heart rate (HR) measured during exposure.

## Materials and Methods

### Study participants.

BP and HR were measured in healthy individuals at 30-min intervals during 2-hr controlled exposures carried out at the Gage Occupational and Environmental Health Unit. Measurements were included from two randomized CAP exposure studies (studies A and B) with identical exposure protocols but different cardiorespiratory outcome measures. We report data for the exposures that were common to both studies, 150 μg/m^3^ CAP+O_3_ and PFA. Twenty-one subjects were included from study A. We have previously reported BP data from this study (Brook et al. 2002), measured immediately before and 10–20 min after CAP+O_3_ exposure, which showed no significant changes in systolic BP (SBP) or diastolic BP (DBP). Two subjects were also included from study B (which included both respiratory and vascular measures in individuals exposed to CAP±O_3_) who had complete data for both exposures. Therefore, 23 individuals (21 from study A and 2 from study B) had both treatments. Exposures were a minimum of 1 day apart, and the median interval between exposures was 1 week. Both studies had prior approval from the human subjects research ethics review committees of the University of Toronto and St. Michael’s Hospital; all participants gave written informed consent before enrollment. Healthy 18- to 50-year-old non-smokers were enrolled who met the following criteria: no cardiovascular disease or diabetes, normotensive (100/50 < BP < 140/90 mm Hg), not using lipid-lowering medications or inhaled/oral corticosteroids, and free of upper respiratory tract infections for at least 3 weeks before exposure testing.

### Exposure protocol.

To reduce the impact of diurnal variation, subjects arrived at the lab around 0900 hr for each exposure. Each subject underwent a vascular and respiratory work-up of approximately 1 hr followed immediately by a 2-hr exposure to either CAP+O_3_ or PFA using a facemask delivery. During exposure, subjects were asked to list any symptoms that they had, and immediately afterward, they were asked if they thought that they were exposed to a pollutant. Subjects were blind to the two exposure treatments. CAP exposures were produced with a high-flow virtual impactor system, using fine PM (PM_2.5_) drawn from outside the laboratory; O_3_ produced by an arc generator was added upstream of the particle concentrator. During PFA exposures, a HEPA filter was inserted inline downstream of the particle concentrator. The human exposure facility and exposure characterization have been described in detail elsewhere (Petrovic et al. 2000; Urch et al. 2004).

### BP and HR measures.

BP and HR are highly variable, and even minor alterations in temperature and physical activity can substantially alter readings (Parati et al. 2003). In order to minimize effects not mediated by air pollution, BP and HR were measured throughout the actual exposure period while subjects were resting quietly and at constant temperature (~ 23°C). Participants were provided with an automated oscillometric BP monitor (Oscar-1; SunTech Medical Instruments, Inc., Raleigh, NC, USA) immediately before entering the exposure enclosure, with the arm-cuff secured on their left upper arm. In addition, a PC-based real-time 12-lead electrocardiogram (ECG; PC-ECG 1200; Norav Medical Ltd., Kiryat Bialik, Israel) was connected. After sitting inside the exposure enclosure, the door was sealed and concentrator pumps turned on. When resting quietly, subjects were asked to place their left forearm on their left leg and then turn on the BP monitor with their right hand (0 hr baseline exposure measure). BP measures were repeated at 30-min intervals during exposure, with the final BP measurement made at 2 hr, immediately before the end of the exposure. HR was determined at 30-min intervals by the 12-lead ECG. The technician recording the BP and HR was blinded to the exposure treatments (CAP+O_3_ vs. PFA).

### Statistical methods.

Intraexposure comparisons of BP (SBP, DBP, mean) and HR outcome variables were made using a 0-hr value as the baseline reference to assess the change over the course of the 2-hr exposure. The respective exposure values at 2 hr were calculated as a linear change (Δ = 2 hr – 0 hr) for each participant. The nonparametric Wilcoxon signed rank test was used to compare the difference in the 0- to 2-hr change between the two exposure treatments and was confirmed using percentage change, as well as the slopes of the individual straight lines fitted over the five 30-min measurements. Further support was provided by Student’s *t*-test of the linear and percentage changes and by a repeated-measures analysis of the linear trend over time. Linear regression analysis was also used to estimate the slope of the relationship between the 2-hr change in BP (SBP, DBP, mean) and the concentration of individual PM constituents (organic and elemental carbon, inorganic ions, and trace elements) as well as the total mass concentration. PM constituent measurements have previously been described (Urch et al. 2004). An unpaired *t*-test was used for interexposure comparisons of the mean PM_2.5_, O_3_, and environmental values. The Wilcoxon signed rank test was used for inter-exposure comparison of the number of symptoms reported during exposure. All analyses were performed using SAS for Windows (release 8.02; SAS Institute Inc., Cary, NC, USA).

## Results

The participants ranged in age from 19 to 50 years, with a mean (± SD) age of 32 ± 10 years, and included 13 males and 10 females (Table 1).

The mean exposure concentration (± SD) of the PM_2.5_ total mass was 147 ± 27 with a range of 102–214 μg/m^3^ (Table 2). The mean O_3_ concentration was less variable, with a mean (± SD) of 121 ± 3 and range of 115–128 ppb. As expected, the PM_2.5_ total mass and O_3_ concentrations were significantly different between exposure treatments (*p* < 0.0001 for both). The exposure concentrations of the other copollutants measured, which included nitrogen oxides, sulfur dioxide, and carbon monoxide, were low and less than measured ambient levels. There were no significant treatment differences for any of these copollutants, for temperature, or for relative humidity.

Symptoms reported by subjects during exposures were few, if any (mean number of symptoms reported ± SD was 1.0 ± 1.9 for CAP+O_3_ vs. 0.5 ± 1.2 for PFA; *p* = 0.039). For example, only 4 of 23 subjects (17%) reported smelling an odor during CAP+O_3_ exposure, compared with 1 during PFA, which is not surprising because the O_3_ generation began at the exposure start (time 0 hr) and progressively increased over the first 10 min, then remained stable at 120 ppb over the duration of the exposure. Subjects were not able to identify the pollutant (CAP+O_3_) exposure with any precision, because only 52% (12 of 23) responded “yes” immediately afterward to the question, “Do you think you were exposed to a pollutant?” After PFA exposure, 30% (7 of 23) responded “yes” they were exposed to a pollutant, including 4 of the 12 who said “yes” after CAP+O_3_.

Table 3 shows the median DBP and SBP values over the course of CAP+O_3_ and PFA exposures. The last column shows the 0- to 2-hr linear change. DBP showed a progressive trend to increase over time during CAP+O_3_ exposure, from a 0-hr baseline, compared with a flat response during PFA. A significant 2-hr increase of 6 mm Hg (binomial 95% CI, 0–11; *p* = 0.013), or 9.3% above the 0-hr value, was observed during CAP+O_3_ exposure. This increase was significantly different (*p* = 0.017, unadjusted for basal BP) from the small, nonsignificant, 1 mm Hg (95% CI, −2 to 4) change observed during PFA. Analyses using percent change and slope as end points, as well as parametric analyses, supported these findings. There were no significant changes of SBP, mean arterial pressure (MAP; 2/3 DBP + 1/3 SBP), or HR during either exposure.

**Figure 1:**
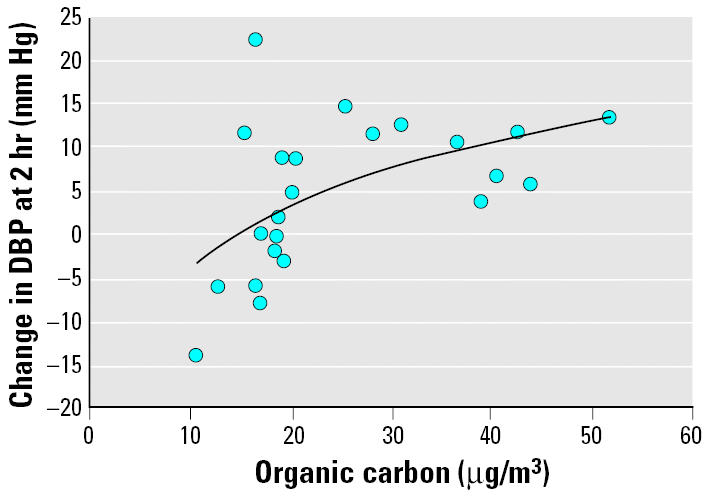
Change in DBP at 2-hr exposure to approximately 150 μg/m^3^ of CAP+O_3_ versus the estimated exposure mass concentration of the organic carbon fraction of CAP (shown as 2-hr – 0-hr linear change). The solid line indicates the regression line. *y* = 10.8 ln(*x*) – 28.8; *r* = 0.53; *p* = 0.009; *n* = 23.

The observed changes in DBP prompted us to look more closely at which constituents of the CAP may be responsible. Figure 1 suggests a nonlinear relationship between the individual 2-hr linear changes in DBP during CAP+O_3_ exposure and the estimated exposure concentration of organic carbon. Indeed, the strongest association was found with the log_e_ (ln) concentration of organic carbon (*r* = 0.53, *p* = 0.009), much stronger, for example, than with PM_2.5_ total mass (*r* = 0.25, *p* = 0.27). For these individuals, the mean total carbon (organic + elemental carbon) fraction of the PM_2.5_ total mass was 28.4 ± 13.3 μg/m^3^ (range, 11.4–56.5), of which the majority, 25.0 ± 11.6 μg/m^3^, was organic carbon. There was also a significant correlation for the 2-hr change in MAP and the ln concentration of organic carbon (*r* = 0.56, *p* = 0.006) but, again, much weaker with total mass (*r* = 0.21, *p* = 0.35). A weaker correlation was shown between SBP and ln organic carbon (*r* = 0.45, *p* = 0.031) but not with total mass (*r* = 0.05, *p* = 0.83).

## Discussion

For individuals exposed to CAP+O_3_ for 2 hr, we observed a significant increase of 6 mm Hg (9.3%) in DBP. A particularly interesting finding is the strong association between the size of the 2-hr DBP change (and MAP) and the carbon content of the fine particles. This result provides additional validity and biologic plausibility to the observed blood pressure increase. The levels of both pollutants in this study, although high, were at environmentally relevant concentrations [U.S. Environmental Protection Agency (EPA) 2002]. These findings shed further light upon the biologic mechanisms that link air pollution exposure to enhanced cardiovascular risk.

The significant correlation between the organic carbon fraction of the PM_2.5_ and BP change suggests that PM composition is an important factor in cardiovascular health effects and that the carbonaceous fraction in particular warrants further study. It is also possible that carbon is only a measurement proxy for the pollutant actually responsible. A source apportionment study of local ambient PM_2.5_, over the same time period, has shown that 40–50% of the organic carbon was motor vehicle related—through either combustion or road dust (Lee et al. 2003). Exposure to urban traffic has been identified as a potential risk factor for cardiovascular disease (Peters and Pope 2002). Of particular note, a recent study reported an association between exposure to urban traffic and the onset of myocardial infarction, as soon as 1 hr afterward (Peters et al. 2004). Although we observed no association between other pollutants (e.g., O_3_) and the change in BP, further studies will be required to clarify the specific pollutant(s) responsible. In particular, studies will need to be performed using concentrated particles with and without added O_3_ to determine if there are important additive or synergistic interactions.

Of substantial interest is that three epidemiologic studies have previously demonstrated associations between air pollution and elevated BP (Ibald-Mulli et al. 2001; Linn et al. 1999; Zanobetti et al. 2004). However, the findings of our study, using a controlled experimental design, greatly strengthen the evidence that air pollution actually plays a causal role in elevating BP.

Several published controlled human exposure studies have not reported a significant increase in DBP after exposures to fine CAP (Gong et al. 2003), fine CAP+O_3_ (Brook et al. 2002), diesel exhaust particles (Nightingale et al. 2000), or O_3_ (Gong et al. 1998). Gong et al. (1998) did, however, report a significant increase in the rate pressure product (SBP × HR) and HR after O_3_ exposure compared with filtered air, although the dose of O_3_ the subjects were exposed to (300 ppb for 3 hr with intermittent exercise, 30–40 L/min) was at least 12 times higher than in our study (120 ppb for 2 hr without exercise). It is probable that other experimental differences at least partially explain the disparity between the present and previous reports. For example, as far as we are aware, the hemodynamic responses (in the aforementioned four controlled human exposure studies) have previously been reported only before and several minutes after exposures. The accurate determination of any pollutant-induced alteration in BP was likely hindered by temperature and pressure changes, physical exertion, and the probable time delay (several minutes) during patient transport from the exposure facility to the site of physiologic measurements. Our present study design minimized some of these confounding variables, by performing repeated measurements of BP during the actual exposure period using an automated oscillometric device while resting stationary in the enclosure.

At present, the precise pathophysiologic mechanisms of the BP increase remain speculative. It is possible that, through activation of lung macrophages or alveolar cells after cellular uptake and/or via interaction with the cell membranes, inhaled pollutants (particles/O_3_) could promote systemic oxidative stress (Donaldson et al. 2003; Kelly 2003). Induction of systemic oxidative stress (Gurgueira et al. 2002; Sorensen et al. 2003) and a pro-inflammatory response (Salvi et al. 1999; Tan et al. 2000) may alter the bioavailability of nitric oxide within the vasculature and/or by increasing several vasoconstrictive factors, such as endothelin (Taniyama and Griendling 2003; Vincent et al. 2001) or asymmetric dimethyl-arginine (Cooke 2000; Dvonch et al. 2004). Indeed, enhanced oxidative stress and systemic inflammation are known to play a key role in the pathophysiology of many cardiovascular diseases, in particular, hypertension (Oparil et al. 2003). An imbalance of the autonomic nervous system favoring an increase in sympathetic drive (Devlin et al. 2003; Gold et al. 2000; Magari et al. 2001) may also contribute to this hypertensive response.

The risks for myocardial infarctions (Peters et al. 2001, 2004), as well as strokes (Hong et al. 2002; Tsai et al. 2003), increase in response to acute elevations in air pollution. These observations suggest that a factor common to the etiology of both adverse outcomes, such as an elevated BP, may play a significant pathophysiologic role. It is well established that relatively small sustained increases in DBP even within the normotensive range, as observed in this study (median change of 6 mm Hg), increase the long-term risk for coronary events and strokes by approximately 30% and 40%, respectively (Lewington et al. 2002; Vasan et al. 2001). However, the cardiovascular risk imposed by the brief vasopressor response observed in this study is less certain. Nevertheless, two hypothetical scenarios may be envisioned. Exposure to high ambient concentrations of air pollutants may initiate a rapid hypertensive response, thus promoting acute cardiovascular events in susceptible individuals. In conjunction, if this vasopressor response continues unabated, gradients in personal exposure to air pollution could contribute to long-term differences in interindividual BP levels. Continued exposure to air pollution could thereby increase the risk for developing chronically elevated BP and possibly overt hypertension.

## Conclusions

We observed a rapid and a statistically significant increase of diastolic BP among individuals exposed to ambient fine particles and O_3_. Lending biologic plausibility to this observation is that the size of the 2-hr change in blood pressure is associated with the carbon content of the fine particles. Although this may help explain the mechanisms whereby air pollution increases cardiovascular risk, the clinical significance of this finding, the responsible pollutants, biologic mechanisms, and the duration of the response require further investigation. Exposure to vehicular traffic in urban centers may provide a common link between our findings and previous studies in which this exposure source was identified as a potential risk factor for cardiovascular disease.

## Figures and Tables

**Table 1:** Participant characteristics (*n* = 23; 13 male and 10 female).

Characteristic	Mean ± SD
Age (years)	32 ± 10
Height (cm)	173 ± 8
Weight (kg)	72 ± 11
SBP (mm Hg)	117 ± 10
DBP (mm Hg)	77 ± 9
HR (beats/min)	70 ± 11

**Table 2:** Particle, O_3_, and environmental exposure measures (mean ± SD; *n* = 23).

Measure	CAP+O_3_	PFA
PM_2.5_ (μg/m^3^)	147 ± 27*	2 ± 2
O_3_ (ppb)	121 ± 3*	8 ± 5
NO_x_ (ppb)	55 ± 18	51 ± 23
SO_2_ (ppb)	3 ± 2	4 ± 5
CO (ppm)	0.6 ± 0.2	0.5 ± 0.2
Temperature (°C)	23.0 ± 1.3	23.3 ± 1.3
Relative humidity (%)	49 ± 8	48 ± 9

NO_x_, nitrogen oxides.

* p < 0.0001, unpaired t-test for CAP+O_3_ versus PFA.

**Table 3:** Median (binomial 95% CI) DBP and SBP (mm Hg) over 2-hr exposures (*n* = 23).

	Exposure time					
	0 hr	0.5 hr	1 hr	1.5 hr	2 hr	2 hr Δ^a^
CAP+O_3_						
DBP	69 (65–75)	73 (68–79)	75 (72–76)	75 (70–76)	78 (71–82)	6 (0–11)*#
SBP	118 (112–127)	117 (111–126)	119 (111–126)	118 (112–124)	121 (113–124)	−1 (–5–4)
PFA						
DBP	74 (69–78)	73 (71–76)	72 (68–78)	76 (70–81)	73 (70–76)	1 (−2–4)
SBP	117 (113–124)	115 (107–121)	118 (110–123)	120 (114–131)	121 (112–123)	0 (−2–5)

a 2-hr - 0-hr linear change.

* p = 0.013 for CAP+O_3_ DBP 2 hr Δ, Wilcoxon signed rank test.

# p = 0.017 for CAP+O_3_ DBP 2 hr Δ versus PFA DBP 2 hr Δ, Wilcoxon signed rank test.
